# Risk factors for mortality of critically ill patients with COVID-19 receiving invasive ventilation

**DOI:** 10.7150/ijms.50039

**Published:** 2021-01-11

**Authors:** Ye Tu, Ping Yang, Yaqun Zhou, Xiaoyan Wen, Qinqin Li, Jing Zhou, Jingjing Wang, Jinqian Hu, Nannan He, Kai Wang, Chaolong Wang, Xuebi Tian, Ailin Luo, Feng Gao

**Affiliations:** 1 Department of Anesthesiology, Tongji Hospital, Tongji Medical College, Huazhong University of Science and Technology, Wuhan, China.; 2 Department of Clinical Laboratory, Tongji Hospital, Tongji Medical College, Huazhong University of Science and Technology, Wuhan, China.; 3 Department of Epidemiology and Biostatistics, School of Public Health, Tongji Medical College, Huazhong University of Science and Technology, Wuhan, China.

**Keywords:** COVID-19, critically ill, mortality, risk factor, invasive ventilation

## Abstract

**Rationale:** Early invasive ventilation may improve outcomes for critically ill patients with COVID-19. The objective of this study is to explore risk factors for 28-day mortality of COVID-19 patients receiving invasive ventilation.

**Methods:** 74 consecutive adult invasively ventilated COVID-19 patients were included in this retrospective study. The demographic and clinical data were compared between survivors and non-survivors, and Cox regression analysis was used to explore risk factors for 28-day mortality. The primary outcome was 28-day mortality after initiation of invasive ventilation. Secondary outcome was the time from admission to intubation.

**Results:** Of 74 patients with COVID-19, the median age was 68.0 years, 53 (71.6%) were male, 47 (63.5%) had comorbidities with hypertension, and diabetes commonly presented. The most frequent symptoms were fever and dyspnea. The median time from hospital admission to intubation was similar in survivors and non-survivors (6.5 days vs. 5.0 days). The 28-day mortality was 81.1%. High Sequential Organ Failure Assessment (SOFA) score (hazard ratio [HR], 1.54; 95% confidence interval [CI], 1.23-1.92; p < 0.001) and longer time from hospital admission to intubation (HR, 2.41; 95% CI, 1.15-5.07; p = 0.020) were associated with 28-day mortality in invasively ventilated COVID-19 patients.

**Conclusions:** The mortality of invasively ventilated COVID-19 patients was particularly striking. Patients with high SOFA score and receiving delayed invasive ventilation were at high risk of mortality.

## Introduction

In December 2019, coronavirus disease 2019 (COVID-19) caused by severe acute respiratory syndrome coronavirus-2 (SARS-CoV-2) first occurred in Wuhan, China. As of November 26, 2020, 60074174 SARS-CoV-2 infections had been reported worldwide. The clinical spectrum of COVID-19 is heterogeneous, ranging from asymptomatic infection to acute hypoxic respiratory failure 1. Among hospitalized patients, 2.3% to 19% required supportive invasive mechanical ventilation as part of their treatments for days to weeks 2-6. Although it was reported that dexamethasone could reduce mortality in invasively ventilated COVID-19 patients 7, owing to the lack of effective agents to date, the timely intubation and invasive mechanical ventilation play a crucial role in countering a progressively oxygen debt and saving life in critically ill COVID-19 patients 8. Determining whether and when to intubate and mechanically ventilate a COVID-19 case with hypoxemic respiratory failure is an intricate decision based on both patient conditions and clinician evaluation, and may have vital implications for individual prognosis. For example, delayed mechanical ventilation is associated with worsened clinical outcomes in ARDS 9. High respiratory drive leading to self-induced lung injury has been suggested as a potential mechanism underlying these observations 10. Correspondingly, utilization of high-flow nasal cannula, which can produce 30-60 L/min of supplemental oxygen and reduce dead space, may cover up the clinical deterioration, therefore delay the time to endotracheal intubation and exacerbate respiratory failure 9. COVID-19 patients presenting with acute respiratory failure were intubated empirically in particular with those manifesting no improvement with non-invasive ventilation, persistent respiratory distress and poor oxygenation (PaO_2_ to FiO_2_ ratio < 150 mmHg) after 2-hours high-flow oxygen therapy or noninvasive ventilation. It may be rational to intubate and mechanically ventilate following the early indications of non-invasive ventilation failure. To date, information about clinical characteristics of patients who required invasive ventilation remains scarce, and risk factors associated with poor outcome of these patients have not been identified, which are of great importance to reduce mortality of critically ill COVID-19 patients.

To address these questions, we carried out a cohort study of invasively ventilated patients with COVID-19. Our primary hypothesis was that longer time from hospital admission to intubation would be associated with increased 28-day mortality. In addition, we explored risk factors associated with 28-day mortality in invasively ventilated COVID-19 patients.

## Methods

### Study design and participants

This single-centered, retrospective, observational study included consecutive patients from Tongji Hospital, Tongji Medical College, Huazhong University of Science and Technology, which was a designated hospital for severe and critically ill patients with COVID-19 in Wuhan, China. From January 10 to February 29, 2020, Adult patients admitted to intensive care unit (ICU) and receiving invasive ventilation were enrolled in this study. COVID-19 was diagnosed according to the World Health Organization (WHO) interim guidance. The study was approved by the Institutional Ethics Board of Tongji Hospital (TJ-IRB20200347). Written informed consent was waived due to the rapid emergence of this infectious disease.

### Data collection

Epidemiological, demographic, clinical, laboratory, radiological, and treatment data were extracted from electronic medical records. All data were checked and reviewed by three physicians (QL, PY, YT). Information recorded included demographic data, comorbidities, symptoms from illness onset to hospital admission, chest computed tomographic (CT) scans, treatments, living status, etc.

Throat swab samples were obtained for SARS-CoV-2 detection using real-time RT-PCR. 200 µL of sample was used for laboratory confirmation of SARS-CoV-2 RNA with RNA isolation kit (Yuesui equipment No.20170583 and 20150302, Da'an gene Co., Ltd, Sun Yat-sen University, China) according to the manufacturer's instructions. A cycle threshold value less than 40 was defined as positive response.

Laboratory values on the day of hospital admission and within 24 hours before endotracheal intubation were recorded, including complete blood count, erythrocyte sedimentation rate, blood biochemical examinations (i.e., liver and renal function, blood glucose, lactate dehydrogenase [LDH], and electrolytes), myocardial enzymes, coagulation profile, cytokine profile, N-terminal pro-B-type natriuretic peptide (NT-proBNP), D-dimer, high sensitivity C-reactive protein (hsCRP), procalcitonin, and ferritin.

### Study outcomes

The primary outcome was 28-day mortality after receipt of invasive mechanical ventilation invasive ventilation. Secondary outcome was the time from hospital admission to intubation.

### Definitions

The disease severity of COVID-19 was defined according to the Chinese management guideline for COVID-19 (Trial Version 7), in which patients with respiratory failure who require mechanical ventilation, or shock, or other organ failure who require intensive care treatment, are classified as critical illness 11. Acute cardiac injury was defined as serum level of high-sensitivity cardiac troponin I (hsTnI) above 99^th^-percentile upper reference limit, regardless of new abnormalities in electrocardiography and echocardiography 12. ARDS was defined according to the Berlin definition 13. Septic shock and sepsis were defined according to the Third International Consensus Definitions for Sepsis and Septic Shock 14. Acute heart failure was defined according to the European Society of Cardiology criteria 15. Acute kidney injury was defined according to the KDIGO clinical practice guidelines 16. Disseminated intravascular coagulation (DIC) was defined according to the JAAM DIC diagnostic criteria 17.

### Statistical analysis

Continuous variables were described as medians and inter-quartile ranges (IQR), and categorical variables as numbers and percentages. The differences between survivors and non-survivors were compared using Mann-Whitney U test, χ² test, or Fisher's exact test, as appropriate. The differences in laboratory findings between hospital admission and before invasive ventilation were compared using Wilcoxon signed-rank test or McNemar's test, as appropriate. For survival analysis, Kaplan-Meier plots were made to visualize the effects of age, cardiac injury and the time from hospital admission to intubation (≥120 h) on 28-day mortality with log-rank test. Univariable and multivariable Cox proportional hazards regression models were used to explore the risk factors associated with 28-day mortality in COVID-19 patients receiving invasive ventilation. Models were adjusted for age, sex and comorbidities. We excluded variables from univariable analysis if their between-group differences were not significant, if the numbers of events were too small to calculate hazard ratios, and if they had collinearity with Sequential Organ Failure Assessment (SOFA) score. The analyses regarding different factors were based on non-missing data, and missing data were not imputed. All statistical analyses were conducted using SPSS version 19.0 and MedCalc software with the level of significance set to α=0.05 (two-tailed).

## Results

### Patient characteristics

109 critically ill patients receiving invasive ventilation were enrolled. After excluding 32 patients without confirmed COVID-19, and 3 patients with incomplete core information in their electronic medical records, 74 patients were included in the final analysis, and 60 (81.1%) of them died at 28 days. As of April 14, 2020, 63 (85.1%) of 74 patients died, 11 (14.9%) extubated and discharged.

Of the 74 patients, the median age was 68.0 years (inter-quartile range [IQR], 61.5-74.0; range, 44.0-87.0 years), and 53 (71.6%) were male. The proportion of non-survivors aged 70 years and older was significantly higher than survivors (37/60 [61.6%] vs. 4/14 [28.6%]; p = 0.036). 47 (63.5%) of 74 patients had one or more comorbidities, with hypertension, cardiovascular disease and diabetes commonly presented. The most frequent symptom was fever (68 [91.9%]), followed by dyspnea (44 [59.5%]), dry cough (43 [58.1%]), fatigue (27 [36.5%]), and diarrhea (24 [32.4%]). Less common symptoms were sputum production, myalgia, headache, nausea/vomiting, and abdominal pain (Table 1). Abnormalities in thoracic CT scans were observed among all of the 74 patients, and typical CT findings included bilateral pulmonary parenchymal ground-glass and consolidative opacities. Of the 74 patients, the median time from symptom onset to hospital admission, the time from hospital admission to intubation, and the time from symptom onset to intubation were 10.5 days (IQR, 7.0-15.3), 6.0 days (IQR, 4.0-10.0), and 18.4 days (IQR, 15.0-24.0) respectively. The time from endotracheal intubation to death was 5.0 (IQR, 3.0-9.0) days in non-survivors. Demographic, clinical, and radiological features did not show significant differences between survivors and non-survivors (Table 1).

### Laboratory findings

On hospital admission, neutrophilia (neutrophil count ≥ 6.3 × 10^9^/L), lymphopenia (lymphocyte count < 0.8 × 10^9^/L), eosinopenia (eosinophil count < 0.02 × 10^9^/L) and hypoalbuminemia (level of albumin < 30 g/L) were detected in 41 (55.4%), 52 (70.3%), 62 (83.8%) and 26 (35.6%) patients, respectively. Increased levels of LDH, D-dimer, hsTnI, and NT-proBNP beyond the normal values were detected in 68 (91.9%), 63 (91.3%), 19 (30.6%), and 49 (84.5%) patients, respectively. Levels of hsCRP, procalcitonin, ferritin, interleukin-2 receptor (IL-2R), and interleukin-6 (IL-6) in most patients were far beyond the normal values. Non-survivors had higher creatine kinase isoenzyme-MB and NT-proBNP than survivors (Table 2).

Before intubation, white blood cell (WBC) and neutrophil counts were increased, and leukocytosis (WBC count ≥ 10×10^9^/L) and increased neutrophils (≥ 6.3×10^9^/L) were more frequent than hospital admission in the 74 patients (Table 3). Compared with survivors, non-survivors had more frequent cardiac injury with increased hsTnI and NT-proBNP, coagulation dysfunction with prolonged prothrombin time and increased international normalized ratio, and remarkably higher Acute Physiology and Chronic Health Evaluation (APACHE) II and SOFA scores before invasive ventilation (Table 3).

### Interventions and complications

Of the 74 patients, 68 (91.9%) patients received antivirals (abidol, 55 [74.3%]; oseltamivir, 19 [25.7%]; lopinavir/ritonavir, 15 [20.3%]; remdesivir/placebo, 2 [2.7%]), and all patients were prescribed with empirical antibiotics (moxifloxacin, 62 [83.8%]; cefoperazone sulbactam, 36 [48.6%]; carbapenem, 12 [16.2%]; levofloxacin, 7 [9.5%]). Additionally, 60 (81.1%) patients received systematic corticosteroids, and albumin therapy was administered to 23 (31.1%) patients. For oxygen therapy, 66 (89.2%) patients received HFNC and 63 (85.1%) received NIV before invasive ventilation. Of the 14 patients who survived, 1 had received extracorporeal membrane oxygenation support. There were no significant differences in pharmacological treatments, prone position ventilation, and continuous renal replacement therapy between survivors and non-survivors.

In terms of complications, all the survivors and non-survivors had ARDS and sepsis. Compared with survivors, more non-survivors experienced acute heart failure (85.0% vs. 14.3%; p < 0.001), acute kidney injury (58.3% vs. 0.0%; p < 0.001), DIC (78.3% vs. 28.5%; p = 0.001), and septic shock (100% vs. 57.1%; p < 0.001).

### Survival analysis and predictors of 28-day mortality

**Figure 1:**
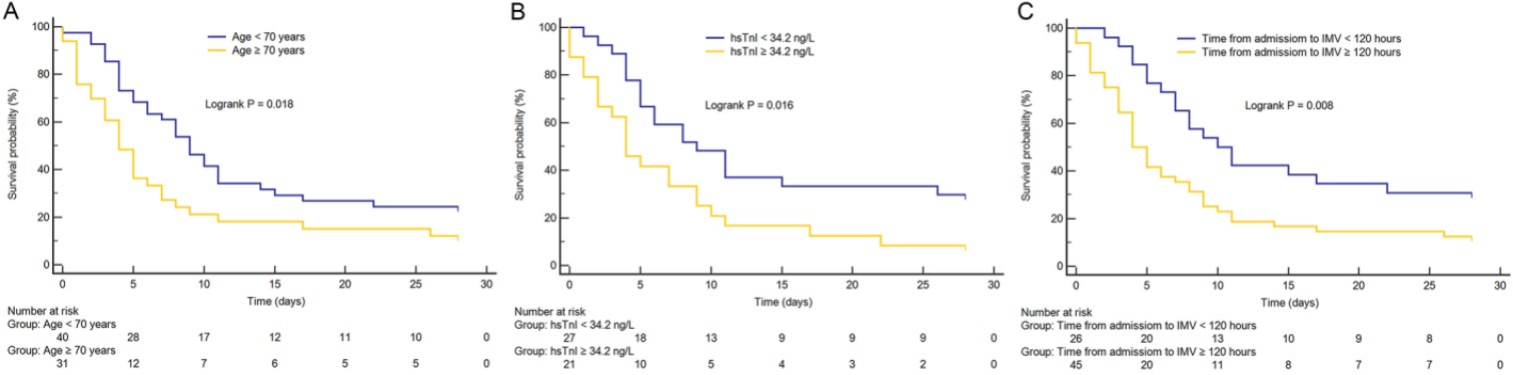
** Kaplan-Meier survival curves.** (A) The impact of age on mortality in critically ill patients with coronavirus disease 2019 (COVID-19). (B) The impact of high-sensitivity cardiac troponin I (hsTnI) level on mortality in critically ill patients with COVID-19. (C) The impact of time from hospital admission to invasive mechanical ventilation (IMV) on mortality in critically ill patients with COVID-19. Groups were compared by using log-rank test. All log-rank *p* value < 0.05. Numbers at risk are indicated.

As shown in Figure 1, Kaplan-Meier survival curves showed that patients with advanced age (≥ 70 years), longer time from hospital admission to intubation (≥ 120 hours), and cardiac injury had higher 28-day mortality than patients with younger age, shorter time from hospital admission to intubation (< 120 hours), and non-cardiac injury (all p < 0.05), respectively.

In univariable Cox proportional hazard regression analysis, advanced age, high SOFA score, longer time from hospital admission to intubation, high hsTnI and LDH levels were associated with death. After adjusting for age, gender and comorbidities, the multivariable model showed that high SOFA score (hazard ratio [HR], 1.54; 95% confidence interval [CI], 1.23-1.92; p < 0.001) and longer time from hospital admission to intubation (≥ 120 hours) (HR, 2.41; 95% CI, 1.15-5.07; p = 0.020) were independent predictors of 28-day mortality in COVID-19 patients receiving invasive ventilation (Table 4).

## Discussion

We reported 74 critically ill cases with COVID-19 receiving invasive ventilation tended to be older, and characterized by cytokine storm and multiple organ dysfunction. 60 (81.1%) of 74 patients died within 28 days after initiation of invasive ventilation. High SOFA score and delayed intubation were associated with 28-day mortality.

Recent studies have demonstrated that survivals among COVID-19 patients with respiratory failure are similar to those in other viral pneumonias and ARDS 18, 19. However, these studies did not analyze the outcomes on the basis of timing of intubation. Consideration for substantial fluctuates in transpulmonary pressures and self-induced lung injury have been proposed as reasons for early intubation in COVID-19 20, 21. In this case, a study would be warranted to address if the timing of intubation influences the clinical outcomes. Our results provide evidence that delayed intubation is substantially associated with 28-day mortality in this vulnerable population of critically ill patients. Noteworthy, these data suggest that strategy for initiating intubation need be adjusted, and early intubation for invasive mechanical ventilation should be applied in critically ill COVID-19 patients. The primary finding of our study is not in accord with the study of Alfonso et al., which reported that timing of intubation was not associated with the risk of death 22. It is requisite to consider that the characteristics, comorbidities, and baseline severity of illness between studies are different. The average SOFA score in the early intubation subgroup in previous study was 10.5, but the median value was 6.0 in present study. These differences in the cohorts reflect the tendency to admit preemptively to ICU for ventilation when necessary, even the condition of COVID-19 patients is slightly deteriorated due to the clinical course. This indicates that our patients were intubated and received invasive ventilation when in a less critical status or in the earlier stage of disease than those in previous study 22. Additionally, the possible explanation is that the definition of early intubation was different between two studies (time from ICU admission to intubation < 8 hours vs. time from hospital admission to intubation ≥120 hours).

In our cohort, 28-day mortality was 81.1%, which was particularly high. Advanced age has been reported to be associated with the severity of SARS-CoV-2 infection, and also an independent predictor of mortality in COVID-19 patients 23-26. In this cohort, invasively ventilated cases were middle-aged and old, with a median age of 68.0 years, confirming that older patients are likely to develop more serious SARS-CoV-2 infection with high mortality. Patients aged 70 and older had shorter time (11.5 days) from illness onset to death than younger patients (20.0 days) 27, indicting a rapid progression of COVID-19 in the elderly. The most fatal complication during SARS-CoV-2 infection was ARDS with an incidence of 100% in this cohort, which was higher than that of 71% in previous study 28. The elderly have been proved to be more likely to develop ARDS than younger patients 29. Only COVID-19 patients had respiratory failure presenting with serious hypoxemia or multiple organ failure are adequately treated with invasive ventilation. This can also explain why the mortality in this cohort was remarkably higher than other studies in which most patients did not require invasive ventilation. Additionally, other possible reasons of high mortality included shortage of medical resources and delayed hospital admission that exist at the early stage of COVID-19 outbreak in Wuhan. We assume that monitoring the progression of SARS-CoV-2 infection in older patients might help physicians make medical decision to reduce the risk of mortality.

Recently, several studies have explored the risk factors for adverse outcomes of COVID-19 23, 30. The SOFA score has been used to evaluate organ dysfunction, and showed significant association with in-hospital mortality in patients with ARDS 31. We found that non-survivors had higher SOFA scores than survivors before invasive ventilation, which was associated with mortality. Hence, high SOFA score could help clinicians to identify at an early stage those patients with COVID-19 who have poor prognosis. Previous study has reported that high SOFA score at hospital admission was a risk factor for death in COVID-19 patients 23. Of the 74 patients in this cohort, leucocytosis, increased neutrophil count and procalcitonin were more common before invasive ventilation, suggesting that secondary bacterial infection might has been developed in a large proportion of patients. More frequent lymphopenia and eosinopenia, as well as increased LDH, hsCRP, NT-proBNP, ferritin, IL-2R, IL-6 and tumor necrosis factor-α, were detected before invasive ventilation, which were similar to previous study 32. These abnormalities of laboratory data indicated invasively ventilated COVID-19 patients commonly experienced severe systemic inflammation and life-threatening conditions, manifested by clinical symptoms and cytokine storm. In this cohort, non-survivors had more fatal complications than survivors after ICU admission. Besides ARDS, most non-survivors eventually developed acute heart failure, acute cardiac injury, acute kidney injury, DIC, and septic shock.

In this cohort, survivors and non-survivors were comparable for clinical features and laboratory findings on hospital admission. The time from symptom onset to hospital admission in survivors and non-survivors were comparable, indicating that patients with different outcomes in our cohort had similar courses of SARS-CoV-2 infection on hospital admission.

Our study has several limitations. Firstly, limited by a small sample size, especially the number of non-survivors, variables with statistical non-significance may not be ruled out. Risk factors for mortality identified in this cohort should be verified in large-sample, multi-center, prospective trails. Secondly, the average time from symptom onset to hospital admission in this cohort was 10.5 days, which indicated delayed hospital admission of patients. Further studies are needed to investigate the optimal timing of endotracheal intubation, which may be decided comprehensively according to the actual conditions of patients.

## Conclusion

The mortality of invasively ventilated COVID-19 patients was rather high. High SOFA score and delayed invasive ventilation were independent predictors of 28-day mortality. Critically ill patients with COVID-19, who had severe hypoxemia and respiratory failure, may benefit from earlier invasive ventilation.

## Figures and Tables

**Table 1:** Demographics and clinical characteristics in patients with COVID-19

	Total (n = 74)	Non-survivors (n = 60)	Survivors (n = 14)	p-value	
**Age, years**	68.0 (61.5-74.0)	69.0 (63.0-74.0)	66.5 (57.8-72.8)	0.365	
18-69 years	33/74 (44.6%)	23/60 (38.3%)	10/14 (71.4%)	0.036	
≥ 70 years	41/74 (55.4%)	37/60 (61.6%)	4/14 (28.6%)	..	
Gender, male	53/74 (71.6%)	43/60 (71.7%)	10/14 (71.4%)	1.000	
**Comorbidities**					
Hypertension	29/74 (39.2%)	25/74 (33.8%)	4/14 (28.6%)	0.545	
Diabetes	14/74 (18.9%)	12/60 (20.0%)	2/14 (14.3%)	1.000	
Cardiovascular disease	13/74 (27.7%)	12/60 (20.0%)	1/14 (7.1%)	0.440	
Cerebrovascular disease	5/74 (6.8%)	4/60 (6.7%)	1/14 (7.1%)	1.000	
Chronic obstructive pulmonary disease	7/74 (9.5%)	6/60	1/14 (7.1%)	1.000	
Chronic kidney disease	1/74 (1.4%)	1/60	0/14 (0.0%)	1.000	
Chronic liver disease	1/74 (1.4%)	1/60	0/14 (0.0%)	1.000	
**Symptoms**					
Fever	68/74 (91.9%)	55/60 (91.7%)	13/14 (92.9%)	1.000	
Dry cough	43/74 (58.1%)	34/60 (56.7%)	9/14 (64.3%)	0.271	
Sputum production	18/74 (24.3%)	17/60 (28.3%)	1/14 (7.1%)	0.165	
Dyspnea	44/74 (59.5%)	35/60 (58.3%)	9/14 (64.3%)	0.167	
Fatigue	27/74 (36.5%)	24/60 (40.0%)	3/14 (21.2%)	0.233	
Diarrhea	24/74 (32.4%)	22/60 (36.7%)	2/14 (14.3%)	0.127	
Myalgia	10/74 (13.5%)	9/60 (15.0%)	1/14 (7.1%)	0.676	
Nausea/vomiting	5/74 (6.8%)	4/60 (6.7%)	1/14 (7.1%)	1.000	
Headache	8/74 (10.8%)	7/60 (11.75)	1/14 (7.1%)	1.000	
Abdominal pain	2/74 (2.7%)	2/60 (3.3%)	0/14 (0.0%)	1.000	
**Vital signs**					
Heart rate, beats per min	90.0 (80.0-102.0)	89.5 (79.3-102.0)	92.0 (82.0-102.0)	0.729	
Respiratory rate, breaths per min	22.5 (20.0-30.0)	23.0 (20.0-30.0)	22.0 (20.0-30.0)	0.801	
Mean arterial pressure, mmHg	99.0 (89.7-107.3)	99.3 (91.3-107.3)	94.7 (86.7-105.7)	0.404	
Time from symptom onset to hospital admission, days	10.5 (7.0-15.3)	10.5 (7.0-14.8)	12.5 (8.3-18.5)	0.283	

Time from hospital admission to invasive ventilation, days	6.0 (4.0-10.0)	6.5 (4.3-10.0)	5.0 (1.5-10.3)	0.225	

Time from symptom onset to invasive ventilation, days	18.4 (15.0-24.0)	18.4 (15.0-23.7)	18.9 (14.0-26.5)	0.836	


Data are median (inter-quartile range) or n (%). COVID-19, coronavirus disease 2019.

**Table 2:** Laboratory findings in patients with COVID-19 on hospital admission

	Total (n = 74)	Non-survivors (n = 60)	Survivors (n = 14)	p-value
**White blood cell count, ×10^9^/L**	8.0 (5.7-11.6)	8.1 (5.9-11.6)	7.5 (3.9-16.9)	0.535
< 4	6/74 (8.1%)	3/60 (5.0%)	3/14 (21.4%)	0.015
4-10	46/74 (62.2%)	42/60 (67.7%)	4/14 (28.6%)	..
≥ 10	22/74 (29.7%)	15/62 (24.2%)	7/14 (50.0%)	..
**Neutrophil count, ×10^9^/L**	7.2 (4.3-11.2)	7.2 (4.7-9.9)	6.1 (2.8-12.3)	0.408
< 1.8	1/74 (1.4%)	0/60 (0.0%)	1/14 (7.1%)	< 0.001
1.8-6.3	32/74 (43.2%)	25/60 (41.7%)	7/14 (50.0%)	..
≥ 6.3	41/74 (55.4%)	35/60 (58.3%)	6/14 (42.9%)	..
**Lymphocyte count, ×10^9^/L**	0.64 (0.44-0.94)	0.64 (0.42-0.95)	0.73 (0.49-0.96)	0.359
< 0.8	52/74 (70.3%)	44/52 (84.6%)	10/14 (71.4%)	0.264
Monocyte count, × 10^9^/L	0.41 (0.29-0.60)	0.39 (0.28-0.60)	0.53 (0.33-0.62)	0.444
**Eosinophil count, × 10^9^/L**	0.00 (0.00-0.01)	0.00 (0.00-0.01)	0.00 (0.00-0.02)	0.712
< 0.02	62/74 (83.8%)	49/60 (81.7%)	13/14 (92.9%)	0.432
Hemoglobin, g/dL	135.0 (124.0-144.0)	135.0 (124.3-144.0)	137.0 (117.8-146.3)	0.907
**Platelet count, × 10^9^/L**	162.0 (133.5-222.0)	160.5 (131.0-222.0)	169.5 (134.5-235.3)	0.760
< 100	10/74 (13.5%)	9/60 (15.0%)	1/14 (7.1%)	0.676
Alanine transaminase, U/L	29.5 (19.0-44.5)	33.0 (19.0-45.5)	22.0 (155-38.8)	0.197
**Aspartate transaminase, U/L**	42.0 (28.0-59.5)	42.5 (30.0-61.5)	34.5 (23.8-58.0)	0.214
> 40	40/74 (54.1%)	35/60 (58.3%)	5/14 (35.7%)	0.126
**Albumin, g/L**	31.6 (29.1-34.6)	31.8 (28.4-34.7)	31.1 (29.7-32.7)	0.801
< 30	26/73 (35.6%)	22/60 (36.7%)	4/13 (30.8%)	0.760
Total bilirubin, μmol/L	13.4 (10.0-19.3)	13.4 (9.7-19.2)	12.9 (10.5-20.2)	0.735
Direct bilirubin, μmol/L	6.2 (4.4-10.5)	5.8 (4.4-10.2)	8.5 (4.9-11.5)	0.266
Indirect bilirubin, μmol/L	6.4 (4.7-9.0)	6.6 (4.9-9.1)	5.4 (3.8-8.7)	0.204
**Lactate dehydrogenase, U/L**	495.5 (416.5-691.8)	530.5 (427.8-721.0)	462.5 (245.8-653.0)	0.086
> 225	68/72 (91.9%)	58/58 (100.0%)	10/14 (71.4%)	0.001
γ-glutamyl transpeptidase, U/L	81.0 (61.5-98.0)	40.5 (26.8-80.0)	50.5 (34.3-80.3)	0.584
Blood urea nitrogen, mmol/L	7.2 (5.3-10.4)	7.5 (5.3-10.4)	6.4 (4.5-10.6)	0.330
**Creatinine, μmol/L**	81.0 (61.5-98.0)	82.0 (59.0-97.0)	74.0 (62.5-107.3)	0.823
> 104	14/73 (19.2%)	11/59 (18.6%)	3/14 (21.4%)	1.000
Potassium, mmol/L	4.33 (3.77-4.74)	4.32 (3.75-4.74)	4.64 (3.79-4.78)	0.474
Sodium, mmol/L	138.4 (134.6-142.6)	139.0 (136.2-143.2)	133.3 (131.8-137.0)	0.001
Calcium, mmol/L	2.01 (1.92-2.09)	2.06 (1.99-2.15)	1.98 (1.95-2.04)	0.006
**Blood glucose, mmol/L**	7.29 (6.36-10.42)	7.29 (6.39-10.38)	7.48 (5.89-13.37)	0.784
> 7.00	32/49 (65.3%)	25/38 (65.8%)	8/14 (57.1%)	0.566
Prothrombin time, seconds	14.8 (13.7-16.3)	14.9 (13.7-16.7)	14.0 (13.2-15.7)	0.181
Activated partial thromboplastin time, seconds	38.4 (34.7-43.3)	38.2 (34.7-43.7)	39.7 (33.7-43.4)	0.887
International normalized ratio	1.15 (1.04-1.30)	1.17 (1.06-1.33)	1.09 (1.01-1.24)	0.190
**D-dimer, μg/mL**	6.8 (1.3-21.0)	7.9 (1.6-21.0)	2.3 (0.5-18.8)	0.130
< 0.5	6/69 (8.7%)	3/56 (5.4%)	3/14 (21.4%)	0.672
0.5-21	37/69 (53.6%)	29/56 (51.8%)	8/14 (57.1%)	..
> 21	26/69 (37.7%)	23/56 (41.1%)	3/14 (21.4%)	..
**High-sensitivity cardiac troponin I, ng/L**	19.2 (8.1-46.2)	22.0 (8.8-53.2)	9.2 (6.1-19.0)	0.058
≥ 34.2	19/62 (30.6%)	18/49 (36.7%)	1/13 (7.7%)	0.050
Myoglobin, ng/mL	133.1 (59.5-199.7)	134.0 (77.9-198.2)	71.0 (41.4-244.1)	0.412
Creatine kinase isoenzyme-MB, ng/ML	1.3 (0.8-2.7)	1.7 (0.9-3.2)	1.0 (0.6-1.4)	0.025
**N-terminal pro-B-type natriuretic peptide, pg/mL**	636.0 (193.0-1373.5)	751.0 (336.5-1661.0)	249.0 (69.3-555.8)	0.004
> 161	49/58 (84.5%)	32/46 (69.6%)	7/12 (58.3%)	0.460
High sensitivity C-reactive protein, pg/mL	75.5 (45.5-146.6)	75.8 (46.0-155.2)	65.8 (22.7-119.9)	0.441
Erythrocyte sedimentation rate, mm/h	32.0 (19.0-63.0)	30.5 (18.3-61.5)	75.0 (32.0-84.0)	0.073
**Procalcitonin, ng/mL**	0.16 (0.10-0.33)	0.16 (0.11-0.33)	0.22 (0.10-0.36)	0.612
< 0.05	3/60 (5.0%)	3/50 (6.0%)	0/10 (0.0%)	0.098
0.05-0.49	48/60 (80.0%)	39/50 (78.0%)	9/10 (90.0%)	..
0.5-1.99	5/60 (8.3%)	5/50 (10.0%)	0/10 (0.0%)	..
≥ 2	4/60 (6.7%)	3/50 (6.0%)	1/10 (10.0%)	..
**Serum ferritin, μg/L**	1598.5 (1064.5-2547.1)	1587.3 (1066.5-2517.6)	1598.5 (225.0-2014.5)	0.860
≥ 400	33/35 (94.4%)	31/32 (96.9%)	2/3 (66.7%)	0.166
**Interleukin-1β, pg/mL**	5.0 (5.0-5.0)	5.0 (5.0-5.0)	5.0 (5.0-7.4)	0.280
≥ 5 pg/mL	6/45 (13.3.0%)	4/37 (10.8%)	2/8 (25.0%)	0.290
**Interleukin-2 receptor, U/L**	1072.0 (829.5-1409.5)	1063.0 (829.5-1409.5)	1087.5 (823.0-1452.8)	0.812
≥ 710	37/45 (82.2%)	30/37 (81.1%)	7/8 (87.5%)	1.000
**Interleukin-6, pg/mL**	40.3 (19.1-123.1)	58.2 (23.5-134.5)	25.8 (10.2-37.1)	0.093
≥ 7	45/45 (100.0%)	37/37 (100.0%)	8/8 (100.0%)	1.000
**Interleukin-8, pg/mL**	37.2 (15.7-63.4)	37.4 (17.2-63.4)	24.9 (10.5-119.5)	0.635
≥ 62 pg/mL	11/45 (24.4%)	9/37 (24.3%)	2/8 (25.0%)	1.000
**Interleukin-10, pg/mL**	11.3 (5.2-17.8)	9.8 (5.4-15.7)	23.8 (7.5-49.1)	0.063
≥ 9.2 pg/mL	25/45 (55.6%)	19/37 (51.4%)	6/8 (75.0%)	0.629
**Tumor necrosis factor-α, pg/mL**	11.2 (7.2-15.3)	9.9 (7.5-13.0)	10.7 (5.8-14.2)	0.850
> 8.1	30/45 (66.7%)	25/37 (67.6%)	5/8 (62.5%)	1.000

Data are median (inter-quartile range) or n (%). COVID-19, coronavirus disease 2019.

**Table 3:** Laboratory findings in patients with COVID-19 before invasive ventilation

	Total (n = 74)	Non-survivors (n = 60)	Survivors (n = 14)	p-value
**White blood cell count, × 10^9^/L**	14.9 (10.6-17.9)	15.1 (11.3-18.3)	10.5 (7.8-17.9)	0.103
< 4	0/66 (0.0%)	0/52 (0.0%)	0/14 (0.0%)	0.015
4-10	13/66 (19.7%)	7/52 (13.4%)	6/14 (42.9%)	..
≥ 10	53/66 (80.3%)	46/52 (88.5%)	7/14 (50.0%)	..
**Neutrophil count, × 10^9^/L**	13.8 (9.7-16.9)	14.1 (10.1-17.0)	9.4 (5.8-16.8)	0.083
< 1.8	1/66 (1.5%)	0/52 (0.0%)	1/14 (7.1%)	< 0.001
1.8-6.3	3/66 (4.5%)	0/52 (0.0%)	3/14 (21.4%)	..
≥ 6.3	62/66 (94.0%)	52/52 (100.0%)	10/14 (71.4%)	..
**Lymphocyte count, × 10^9^/L**	0.50 (0.37-0.72)	0.49 (0.36-0.69)	0.68 (0.37-0.79)	0.265
< 0.8	55/66 (83.3%)	44/52 (84.6%)	11/14 (78.6%)	0.688
Monocyte count, × 10^9^/L	0.46 (0.34-0.63)	0.46 (0.34-0.64)	0.46 (0.28-0.62)	0.748
**Eosinophil count, × 10^9^/L**	0.00 (0.00-0.05)	0.00 (0.00-0.07)	0.00 (0.00-0.08)	1
< 0.02	46/66 (69.7%)	36/52 (69.2%)	10/14 (71.4%)	1.000
Hemoglobin, g/dL	127.5 (118.8-135.0)	127.0 (119.5-134.8)	130.5 (115.0-142.0)	0.632
**Platelet count, × 10^9^/L**	160.0 (98.0.0-222.3)	160.0 (81.0-221.8)	169.5 (128.7-258.8)	0.308
< 100	17/66 (25.8%)	15/52 (28.8%)	2/14 (14.3%)	0.327
Alanine transaminase, U/L	27.0 (17.0-45.0)	27.0 (17.0-47.0)	27.0 (18.3-45.3)	0.969
**Aspartate transaminase, U/L**	32.5 (21.0-44.8)	26.0 (19.0-37.0)	28.0 (21.5-53.8)	0.962
> 40	22/66 (33.3%)	18/52 (34.6%)	4/14 (28.6%)	0.529
**Albumin, g/L**	29.5 (25.3-33.1)	29.3 (25.2-33.1)	30.5 (27.9-33.2)	0.458
< 30	33/65 (50.8%)	27/52 (51.9%)	6/13 (46.2%)	0.764
Total bilirubin, μmol/L	16.7 (12.0-22.7)	17.1 (12.6-22.7)	14.2 (9.2-20.7)	0.234
Direct bilirubin, μmol/L	7.9 (6.2-11.3)	8.2 (6.2-11.2)	7.2 (4.7-12.1)	0.43
Indirect bilirubin, μmol/L	7.7 (5.4-10.5)	7.7 (5.8-10.8)	6.3 (3.3-9.5)	0.165
**Lactate dehydrogenase, U/L**	595.5 (431.8-863.3)	608.0 (426.8-926.8)	477.0 (396.8-688.0)	0.132
> 225	64/66 (97.0%)	51/52 (98.1%)	13/14 (92.9%)	0.382
γ-glutamyl transpeptidase, U/L	50.0 (35.0-81.0)	49.0 (31.5-75.5)	65.5 (38.3-93.0)	0.342
Blood urea nitrogen, mmol/L	8.0 (5.4-11.4)	8.7 (5.6-12.3)	5.4 (4.8-10.5)	0.051
**Creatinine, μmol/L**	67.0 (55.0-87.0)	67.0 (57.5-87.5)	69.0 (50.3-87.3)	0.671
> 104	9/67 (13.4%)	7/53 (13.2%)	2/14 (14.3%)	1.000
Potassium, mmol/L	4.45 (3.85-4.80)	4.46 (3.88-4.81)	4.19 (3.62-4.89)	0.54
Sodium, mmol/L	139.9 (134.4-144.0)	141.3 (136.1-144.6)	133.0 (129.8-137.3)	< 0.001
Calcium, mmol/L	2.01 (1.92-2.09)	2.01 (1.93-2.09)	2.00 (1.91-2.09)	0.787
**Blood glucose, mmol/L**	9.07 (6.54-11.81)	7.86 (6.36-11.30)	9.95 (6.80-14.34)	0.458
> 7.00	32/49 (65.3%)	25/38 (67.8%)	7/11 (63.6%)	1.000
Prothrombin time, seconds	15.7 (14.9-17.3)	16.0 (15.0-18.1)	15.0 (13.6-16.2)	0.016
Activated partial thromboplastin time, seconds	37.8 (34.4-43.2)	37.9 (35.0-42.9)	37.1 (32.2-44.2)	0.641
International normalized ratio	1.24 (1.14-1.38)	1.25 (1.16-1.45)	1.18 (1.05-1.31)	0.046
**D-dimer, μg/mL**	14.3 (4.0-21.0)	15.6 (6.0-21.0)	8.2 (1.8-21.0)	0.192
< 0.5	1/55 (38.2%)	1/42 (2.4%)	0/13 (0.0%)	0.672
0.5-21	33/55 (60.0%)	24/42 (57.1%)	9/13 (69.2%)	..
≥ 21	21/55 (1.8%)	17/42 (40.4%)	4/13 (30.8%)	..
**High-sensitivity cardiac troponin I, ng/L**	32.3 (13.7-232.6)	43.8 (15.1-303.1)	16.5 (8.7-36.0)	0.044
≥ 34.2	26/51 (51.0%)	22/41 (53.7%)	4/10 (40.0%)	0.499
Myoglobin, ng/mL	86.9 (57.3-186.1)	89.1 (59.4-189.4)	73.8 (49.1-164.6)	0.409
Creatine kinase isoenzyme-MB, ng/ML	2.0 (1.2-4.0)	2.2 (1.2-4.6)	1.9 (1.1-2.1)	0.264
**N-terminal pro-B-type natriuretic peptide, pg/mL**	590.0 (306.0-2032.5)	793.0 (399.3-2608.0)	427.0 (195.0-771.0)	0.044
> 161	46/49 (93.9%)	37/38 (97.4%)	9/11 (81.8%)	0.629
High sensitivity C-reactive protein, pg/mL	68.2 (37.4-152.1)	76.3 (32.9-152.1)	66.6 (43.3-167.3)	0.736
Erythrocyte sedimentation rate, mm/h	50.0 (16.0-65.5)	48.0 (15.5-66.3)	50.0 (20.5-65.5)	0.916
**Procalcitonin, ng/mL**	0.23 (0.11-0.59)	0.24 (0.13-0.71)	0.18 (0.09-0.33)	0.145
< 0.05	0/33 (0.0%)	0/24 (0.0%)	0/9 (0.0%)	0.098
0.05-0.49	24/33 (21.2%)	15/24 (62.5%)	9/9 (100.0%)	..
0.5-1.99	7/33 (72.7%)	7/24 (29.2%)	0/9 (10.8%)	..
≥ 2	2/33 (6.1%)	2/24 (8.3%)	0/9 (0.0%)	..
**Serum ferritin, μg/L**	1328.5 (1022.7-2399.0)	1304.0 (1135.3-1866.9)	2360.9 (386.1-3197.7)	0.730
≥ 400	17/18 (94.4%)	13/13 (100.0%)	4/5 (80.0%)	0.278
**Interleukin-1β, pg/mL**	5.0 (5.0-7.1)	5.0 (5.0-7.7)	5.7 (5.0-6.8)	0.800
≥ 5 pg/mL	16/33 (48.5%)	10/24 (41.7%)	6/9 (66.7%)	0.259
**Interleukin-2 receptor, U/L**	1049.0 (822.5-1484.0)	1049.0 (716.0-1550.0)	1048.5 (886.3-1381.8)	0.710
≥ 710	27/33 (81.8%)	18/23 (78.3%)	9/10 (90.0%)	0.640
**Interleukin-6, pg/mL**	58.9 (27.2-172.3)	68.3 (37.2-173.4)	26.8(14.1-89.2)	0.021
≥ 7	33/33 (100.0%)	23/23 (100.0%)	10/10 (100.0%)	1.000
**Interleukin-8, pg/mL**	40.3 (25.0-74.5)	54.2 (25.9-101.0)	28.3 (13.5-34.0)	0.050
≥ 62	16/33 (30.3%)	14/23 (39.1%)	2/10 (20.0%)	0.453
**Interleukin-10, pg/mL**	11.3 (5.2-17.8)	11.3 (5.3-16.8)	10.8 (5.0-14.0)	0.984
≥ 9.2	18/33 (54.5%)	13/23 (56.5%)	5/10 (50.0%)	0.730
**Tumor necrosis factor-α, pg/mL**	11.2 (7.2-15.3)	11.2 (7.7-15.5)	11.3 (7.1-14.0)	0.754
> 8.1	23/33 (69.7%)	17/23 (73.9%)	6/10 (60.4%)	0.444
APACHE II score	17.0 (15.0-20.0)	19.0 (16.0-21.0)	14.0 (12.0-18.0)	< 0.001
SOFA score	6.0 (5.0-7.0)	6.0 (5.0-8.0)	5.0 (4.0-6.0)	0.001

Data are median (inter-quartile range) or n (%). COVID-19, coronavirus disease 2019; APACHE, Acute Physiology and Chronic Health Evaluation; SOFA, Sequential Organ Failure Assessment.

**Table 4:** Univariate and multivariate Cox regression analysis

	Univariable		Multivariable		
	HR (95% CI)	*p*-value	HR (95% CI)	*p*-value	
Age ≥ 70 years vs. < 70 years	1.80 (1.08-2.99)	0.025			
Gender, male	0.99 (0.57-1.73)	0.982			
Hypertension	1.38 (0.83-2.32)	0.219			
Diabetes	1.22 (0.65-2.31)	0.537			
Time from hospital admission to invasive ventilation ≥ 120 hours vs. < 120 hours	2.03 (1.16-3.53)	0.013	2.41 (1.15-5.07)	0.020	

**Comorbidities**	..	0.332			
1	1.28 (0.70-2.33)	0.422			
2	1.26 (0.59-2.68)	0.555			
3	2.26 (0.95-5.37)	0.066			
White blood cell × 10^9^/L	1.02 (0.99-1.05)	0.217			
Lymphocyte × 10^9^/L	0.93 (0.38-2.28)	0.868			
Lactate dehydrogenase, U/L	1.00 (1.00-1.00)	0.048			
Prothrombin time, Seconds	1.03 (0.98-1.09)	0.273			
High-sensitivity cardiac troponin I, ≥ 34.2 ng/L vs. < 34.2 ng/L	2.04 (1.10-3.80)	0.024			
		
N-terminal pro-B-type natriuretic peptide, pg/mL	1.00 (1.00-1.00)	0.392			
D-dimer	1.02 (0.98-1.06)	0.326			
Interleukin-6	1.00 (1.00-1.00)	0.063			
SOFA score	1.34 (1.16-1.56)	<0.001	1.54 (1.23-1.92)	<0.001	

HR, hazard ratio; CI, confidence interval; SOFA, Sequential Organ Failure Assessment.

## Abbreviations

COVID-19: Coronavirus disease 2019
SARS-CoV-2: severe acute respiratory syndrome coronavirus-2
HFNC: high flow nasal cannula
NIV: non-invasive ventilation
SOFA: High Sequential Organ Failure Assessment
ARDS: acute respiratory distress syndrome
ICU: intensive care unit
CT: Computerized tomography
WBC: white blood cell
LDH: lactate dehydrogenase
NT-proBNP: N-terminal pro-B-type natriuretic peptide
hsTnI: high-sensitivity cardiac troponin I
